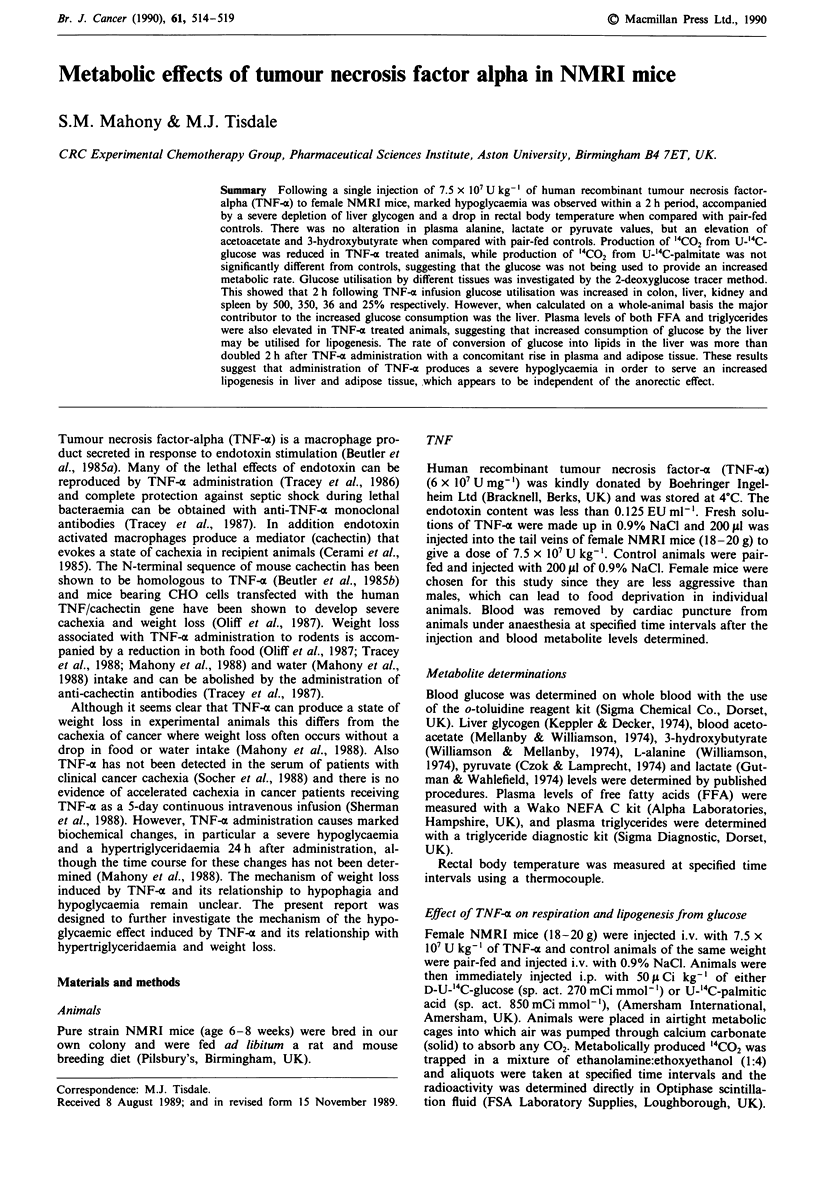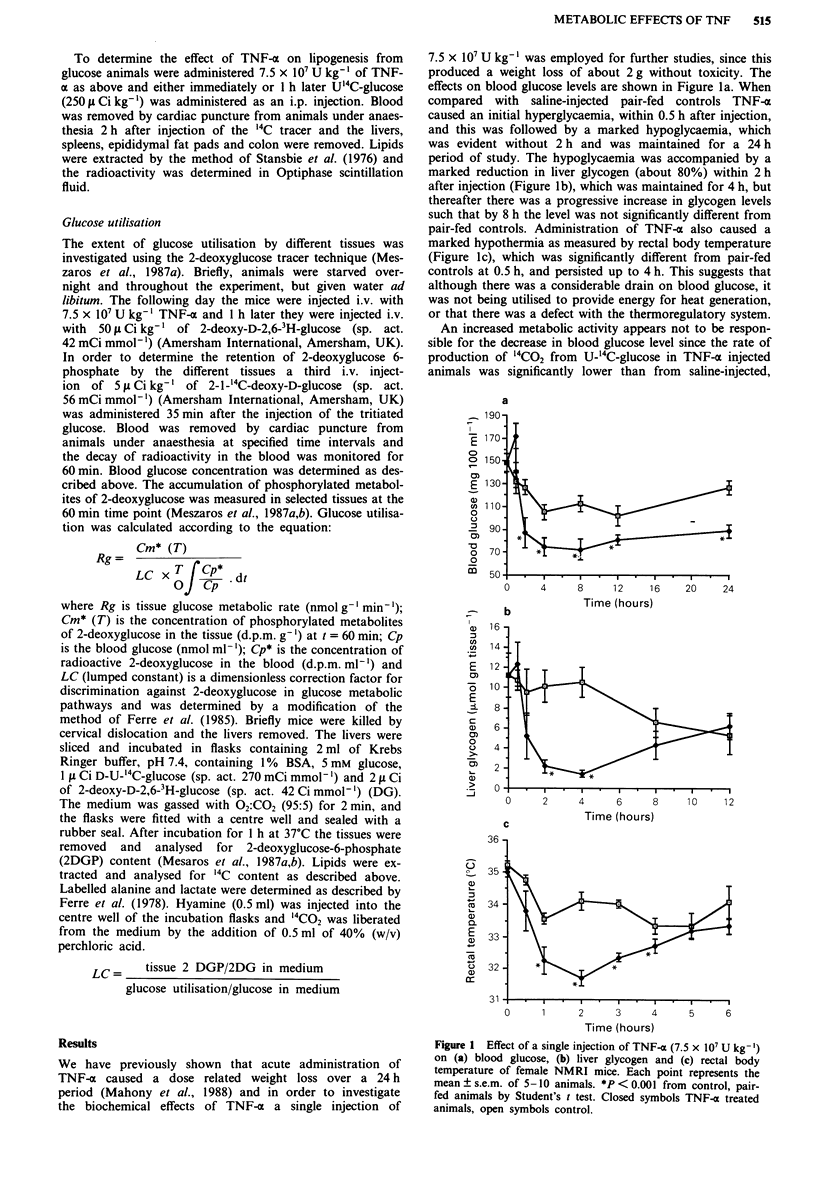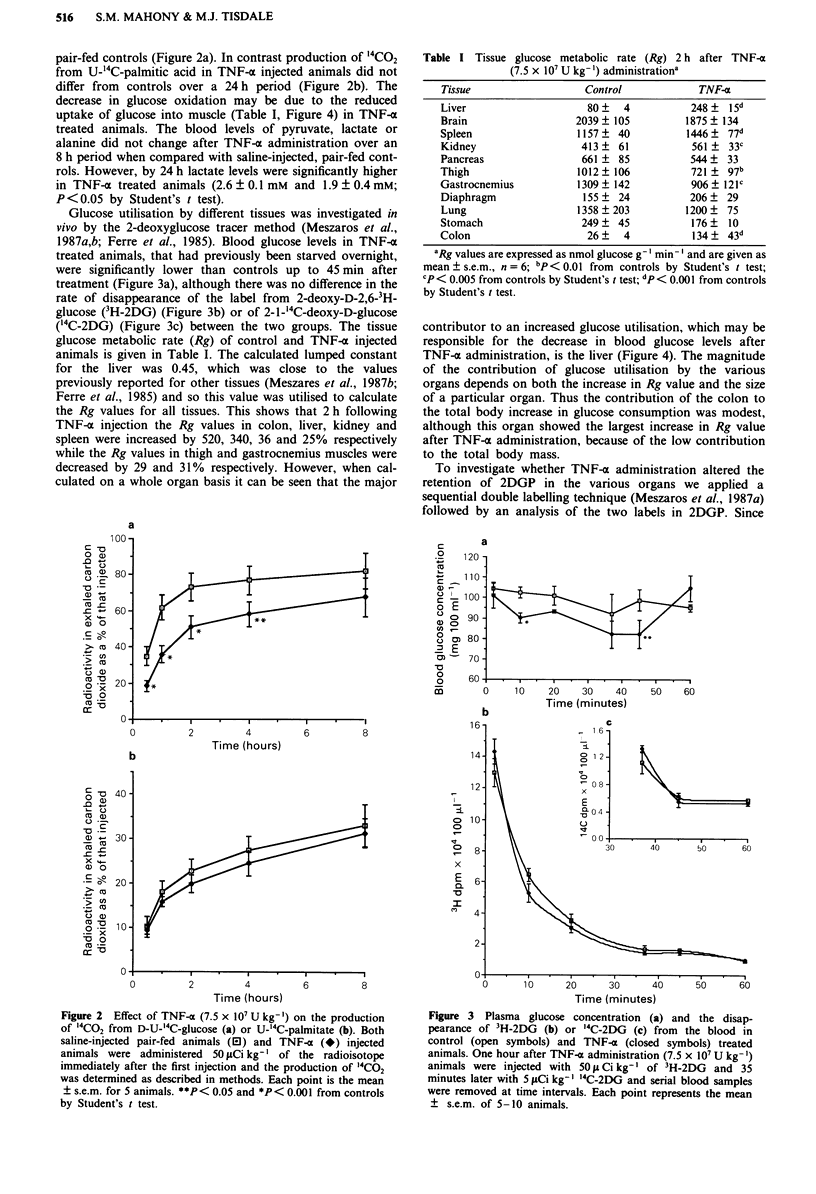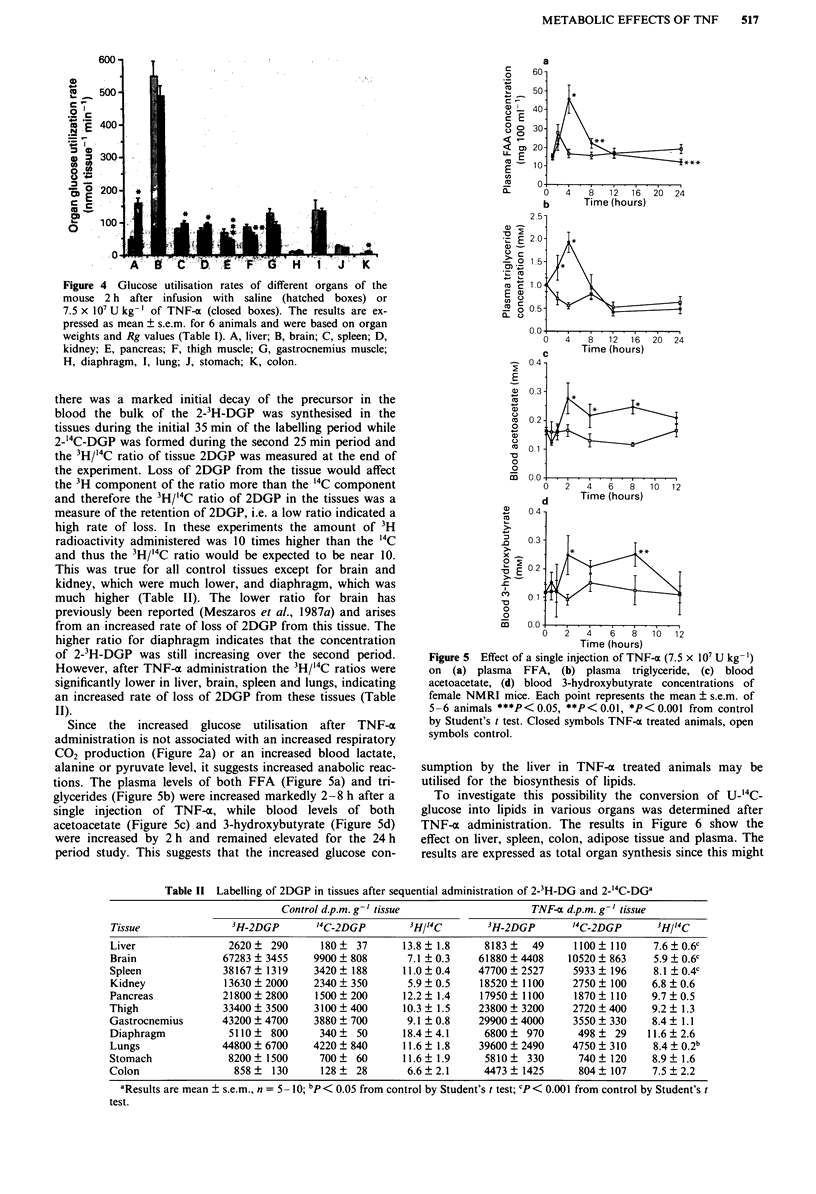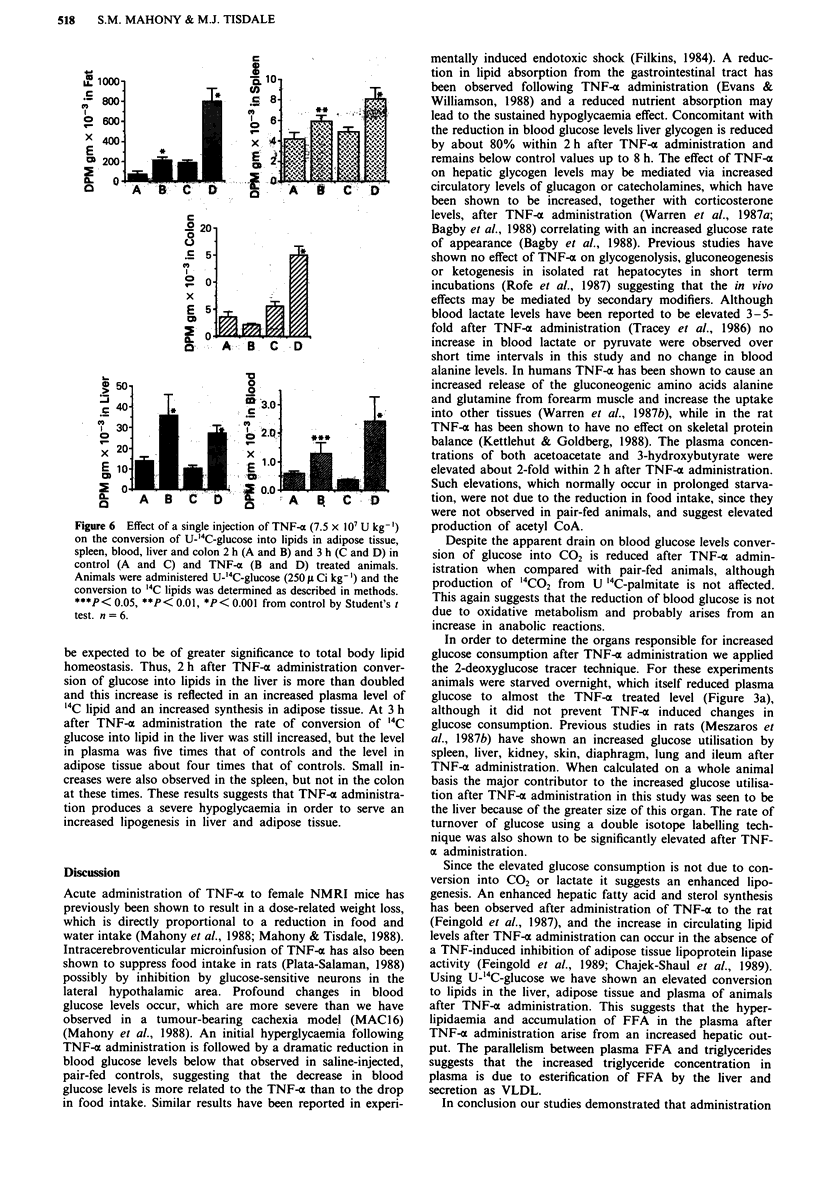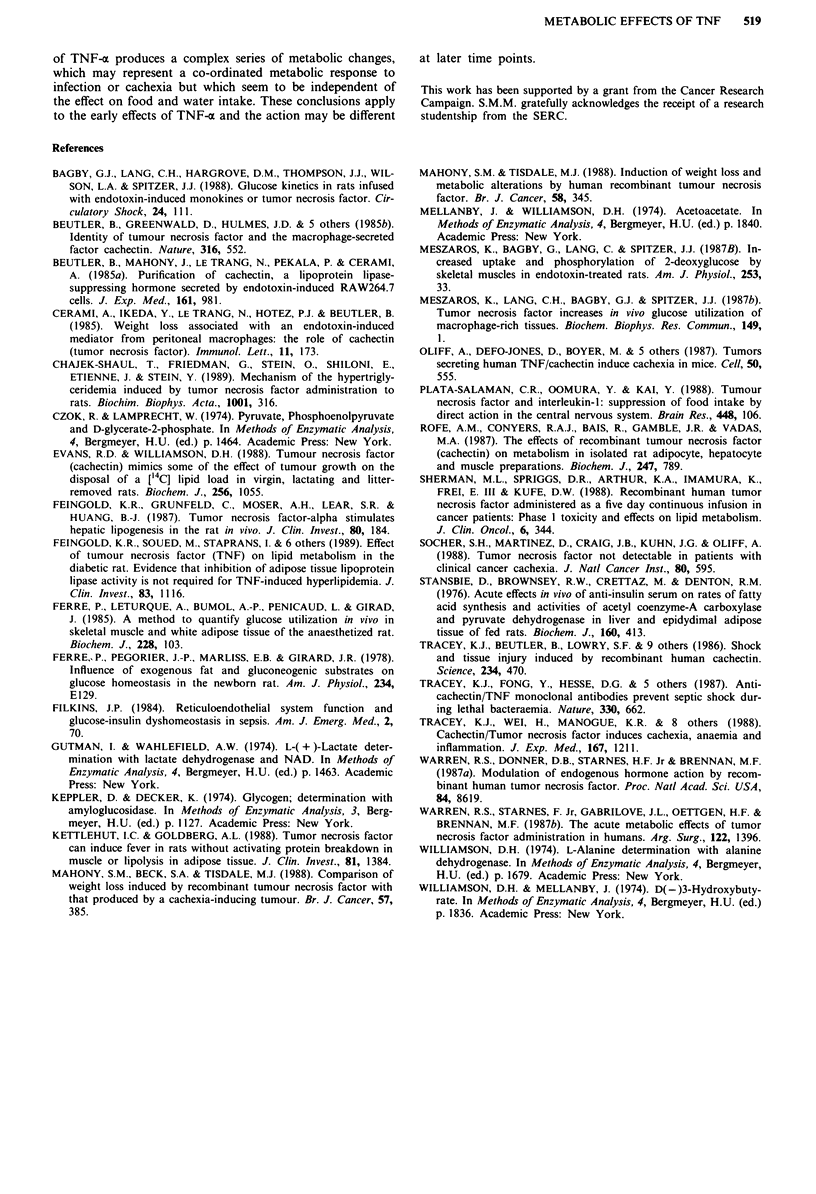# Metabolic effects of tumour necrosis factor alpha in NMRI mice.

**DOI:** 10.1038/bjc.1990.116

**Published:** 1990-04

**Authors:** S. M. Mahony, M. J. Tisdale

**Affiliations:** Pharmaceutical Sciences Institute, Aston University, Birmingham, UK.

## Abstract

Following a single injection of 7.5 x 10(7) U kg-1 of human recombinant tumour necrosis factor-alpha (TNF-alpha) to female NMRI mice, marked hypoglycaemia was observed within a 2 h period, accompanied by a severe depletion of liver glycogen and a drop in rectal body temperature when compared with pair-fed controls. There was no alteration in plasma alanine, lactate or pyruvate values, but an elevation of acetoacetate and 3-hydroxybutyrate when compared with pair-fed controls. Production of 14CO2 from U-14C-glucose was reduced in TNF-alpha treated animals, while production of 14CO2 from U-14C-palmitate was not significantly different from controls, suggesting that the glucose was not being used to provide an increased metabolic rate. Glucose utilisation by different tissues was investigated by the 2-deoxyglucose tracer method. This showed that 2 h following TNF-alpha infusion glucose utilisation was increased in colon, liver, kidney and spleen by 500, 350, 36 and 25% respectively. However, when calculated on a whole-animal basis the major contributor to the increased glucose consumption was the liver. Plasma levels of both FFA and triglycerides were also elevated in TNF-alpha treated animals, suggesting that increased consumption of glucose by the liver may be utilised for lipogenesis. The rate of conversion of glucose into lipids in the liver was more than doubled 2 h after TNF-alpha administration with a concomitant rise in plasma and adipose tissue. These results suggest that administration of TNF-alpha produces a severe hypoglycaemia in order to serve an increased lipogenesis in liver and adipose tissue, which appears to be independent of the anorectic effect.


### Metabolic effects of tumour necrosis factor alpha in NMRI mice

S.M. Mahony & M.J. Tisdale

CRC Experimental Chemotherapy Group, Pharmaceutical Sciences Institute, Aston University, Birmingham B4 7ET, UK.

**Summary** Following a single injection of $7.5 \times 10^7$ U kg$^{-1}$ of human recombinant tumour necrosis factor-alpha (TNF-α) to female NMRI mice, marked hypoglycaemia was observed within a 2 h period, accompanied by a severe depletion of liver glycogen and a drop in rectal body temperature when compared with pair-fed controls. There was no alteration in plasma alanine, lactate or pyruvate values, but an elevation of acetoacetate and 3-hydroxybutyrate when compared with pair-fed controls. Production of $^{14}CO_2$ from U-$^{14}$C-glucose was reduced in TNF-α treated animals, while production of $^{14}CO_2$ from U-$^{14}$C-palmitate was not significantly different from controls, suggesting that the glucose was not being used to provide an increased metabolic rate. Glucose utilisation by different tissues was investigated by the 2-deoxyglucose tracer method. This showed that 2 h following TNF-α infusion glucose utilisation was increased in colon, liver, kidney and spleen by 500, 350, 36 and 25% respectively. However, when calculated on a whole-animal basis the major contributor to the increased glucose consumption was the liver. Plasma levels of both FFA and triglycerides were also elevated in TNF-α treated animals, suggesting that increased consumption of glucose by the liver may be utilised for lipogenesis. The rate of conversion of glucose into lipids in the liver was more than doubled 2 h after TNF-α administration with a concomitant rise in plasma and adipose tissue. These results suggest that administration of TNF-α produces a severe hypoglycaemia in order to serve an increased lipogenesis in liver and adipose tissue, which appears to be independent of the anorectic effect.

Tumour necrosis factor-alpha (TNF-α) is a macrophage product secreted in response to endotoxin stimulation (Beutler *et al.*, 1985*a*). Many of the lethal effects of endotoxin can be reproduced by TNF-α administration (Tracey *et al.*, 1986) and complete protection against septic shock during lethal bacteraemia can be obtained with anti-TNF-α monoclonal antibodies (Tracey *et al.*, 1987). In addition endotoxin activated macrophages produce a mediator (cachectin) that evokes a state of cachexia in recipient animals (Cerami *et al.*, 1985). The N-terminal sequence of mouse cachectin has been shown to be homologous to TNF-α (Beutler *et al.*, 1985*b*) and mice bearing CHO cells transfected with the human TNF/cachectin gene have been shown to develop severe cachexia and weight loss (Oliff *et al.*, 1987). Weight loss associated with TNF-α administration to rodents is accompanied by a reduction in both food (Oliff *et al.*, 1987; Tracey *et al.*, 1988; Mahony *et al.*, 1988) and water (Mahony *et al.*, 1988) intake and can be abolished by the administration of anti-cachectin antibodies (Tracey *et al.*, 1987).

Although it seems clear that TNF-α can produce a state of weight loss in experimental animals this differs from the cachexia of cancer where weight loss often occurs without a drop in food or water intake (Mahony *et al.*, 1988). Also TNF-α has not been detected in the serum of patients with clinical cancer cachexia (Socher *et al.*, 1988) and there is no evidence of accelerated cachexia in cancer patients receiving TNF-α as a 5-day continuous intravenous infusion (Sherman *et al.*, 1988). However, TNF-α administration causes marked biochemical changes, in particular a severe hypoglycaemia and a hypertriglyceridaemia 24 h after administration, although the time course for these changes has not been determined (Mahony *et al.*, 1988). The mechanism of weight loss induced by TNF-α and its relationship to hypophagia and hypoglycaemia remain unclear. The present report was designed to further investigate the mechanism of the hypoglycaemic effect induced by TNF-α and its relationship with hypertriglyceridaemia and weight loss.

### Materials and methods

### Animals

Pure strain NMRI mice (age 6-8 weeks) were bred in our own colony and were fed *ad libitum* a rat and mouse breeding diet (Pilsbury's, Birmingham, UK).

Correspondence: M.J. Tisdale.
Received 8 August 1989; and in revised form 15 November 1989.

### TNF

Human recombinant tumour necrosis factor-α (TNF-α) ($6 \times 10^7$ U mg$^{-1}$) was kindly donated by Boehringer Ingelheim Ltd (Bracknell, Berks, UK) and was stored at 4°C. The endotoxin content was less than 0.125 EU ml$^{-1}$. Fresh solutions of TNF-α were made up in 0.9% NaCl and 200 μl was injected into the tail veins of female NMRI mice (18-20 g) to give a dose of $7.5 \times 10^7$ U kg$^{-1}$. Control animals were pair-fed and injected with 200 μl of 0.9% NaCl. Female mice were chosen for this study since they are less aggressive than males, which can lead to food deprivation in individual animals. Blood was removed by cardiac puncture from animals under anaesthesia at specified time intervals after the injection and blood metabolite levels determined.

### Metabolite determinations

Blood glucose was determined on whole blood with the use of the *o*-toluidine reagent kit (Sigma Chemical Co., Dorset, UK). Liver glycogen (Keppler & Decker, 1974), blood acetoacetate (Mellanby & Williamson, 1974), 3-hydroxybutyrate (Williamson & Mellanby, 1974), L-alanine (Williamson, 1974), pyruvate (Czok & Lamprecht, 1974) and lactate (Gutman & Wahlefield, 1974) levels were determined by published procedures. Plasma levels of free fatty acids (FFA) were measured with a Wako NEFA C kit (Alpha Laboratories, Hampshire, UK), and plasma triglycerides were determined with a triglyceride diagnostic kit (Sigma Diagnostic, Dorset, UK).

Rectal body temperature was measured at specified time intervals using a thermocouple.

### Effect of TNF-α on respiration and lipogenesis from glucose

Female NMRI mice (18-20 g) were injected i.v. with $7.5 \times 10^7$ U kg$^{-1}$ of TNF-α and control animals of the same weight were pair-fed and injected i.v. with 0.9% NaCl. Animals were then immediately injected i.p. with 50 μCi kg$^{-1}$ of either D-U-$^{14}$C-glucose (sp. act. 270 mCi mmol$^{-1}$) or U-$^{14}$C-palmitic acid (sp. act. 850 mCi mmol$^{-1}$), (Amersham International, Amersham, UK). Animals were placed in airtight metabolic cages into which air was pumped through calcium carbonate (solid) to absorb any $CO_2$. Metabolically produced $^{14}CO_2$ was trapped in a mixture of ethanolamine:ethoxyethanol (1:4) and aliquots were taken at specified time intervals and the radioactivity was determined directly in Optiphase scintillation fluid (FSA Laboratory Supplies, Loughborough, UK).

To determine the effect of TNF-α on lipogenesis from glucose animals were administered 7.5 × $10^7$ U $kg^{-1}$ of TNF-α as above and either immediately or 1 h later U$^{14}$C-glucose (250 μ Ci $kg^{-1}$) was administered as an i.p. injection. Blood was removed by cardiac puncture from animals under anaesthesia 2 h after injection of the $^{14}$C tracer and the livers, spleens, epididymal fat pads and colon were removed. Lipids were extracted by the method of Stansbie *et al.* (1976) and the radioactivity was determined in Optiphase scintillation fluid.

### *Glucose utilisation*

The extent of glucose utilisation by different tissues was investigated using the 2-deoxyglucose tracer technique (Meszaros *et al.*, 1987*a*). Briefly, animals were starved overnight and throughout the experiment, but given water *ad libitum*. The following day the mice were injected i.v. with 7.5 × $10^7$ U $kg^{-1}$ TNF-α and 1 h later they were injected i.v. with 50 μ Ci $kg^{-1}$ of 2-deoxy-D-2,6-$^3$H-glucose (sp. act. 42 mCi $mmol^{-1}$) (Amersham International, Amersham, UK). In order to determine the retention of 2-deoxyglucose 6-phosphate by the different tissues a third i.v. injection of 5 μ Ci $kg^{-1}$ of 2-1-$^{14}$C-deoxy-D-glucose (sp. act. 56 mCi $mmol^{-1}$) (Amersham International, Amersham, UK) was administered 35 min after the injection of the tritiated glucose. Blood was removed by cardiac puncture from animals under anaesthesia at specified time intervals and the decay of radioactivity in the blood was monitored for 60 min. Blood glucose concentration was determined as described above. The accumulation of phosphorylated metabolites of 2-deoxyglucose was measured in selected tissues at the 60 min time point (Meszaros *et al.*, 1987*a*,*b*). Glucose utilisation was calculated according to the equation:

$$Rg = \frac{Cm^* \ (T)}{LC \times \int_0^T \frac{Cp^*}{Cp} \, dt}$$

where $Rg$ is tissue glucose metabolic rate (nmol $g^{-1}$ $min^{-1}$); $Cm^*$ $(T)$ is the concentration of phosphorylated metabolites of 2-deoxyglucose in the tissue (d.p.m. $g^{-1}$) at $t = 60$ min; $Cp$ is the blood glucose (nmol $ml^{-1}$); $Cp^*$ is the concentration of radioactive 2-deoxyglucose in the blood (d.p.m. $ml^{-1}$) and $LC$ (lumped constant) is a dimensionless correction factor for discrimination against 2-deoxyglucose in glucose metabolic pathways and was determined by a modification of the method of Ferre *et al.* (1985). Briefly mice were killed by cervical dislocation and the livers removed. The livers were sliced and incubated in flasks containing 2 ml of Krebs Ringer buffer, pH 7.4, containing 1% BSA, 5 mM glucose, 1 μ Ci D-U-$^{14}$C-glucose (sp. act. 270 mCi $mmol^{-1}$) and 2 μ Ci of 2-deoxy-D-2,6-$^3$H-glucose (sp. act. 42 Ci $mmol^{-1}$) (DG). The medium was gassed with $O_2$:$CO_2$ (95:5) for 2 min, and the flasks were fitted with a centre well and sealed with a rubber seal. After incubation for 1 h at 37°C the tissues were removed and analysed for 2-deoxyglucose-6-phosphate (2DGP) content (Mesaros *et al.*, 1987*a*,*b*). Lipids were extracted and analysed for $^{14}$C content as described above. Labelled alanine and lactate were determined as described by Ferre *et al.* (1978). Hyamine (0.5 ml) was injected into the centre well of the incubation flasks and $^{14}CO_2$ was liberated from the medium by the addition of 0.5 ml of 40% (w/v) perchloric acid.

$$LC = \frac{\text{tissue 2 DGP/2DG in medium}}{\text{glucose utilisation/glucose in medium}}$$

### Results

We have previously shown that acute administration of TNF-α caused a dose related weight loss over a 24 h period (Mahony *et al.*, 1988) and in order to investigate the biochemical effects of TNF-α a single injection of 7.5 × $10^7$ U $kg^{-1}$ was employed for further studies, since this produced a weight loss of about 2 g without toxicity. The effects on blood glucose levels are shown in Figure 1a. When compared with saline-injected pair-fed controls TNF-α caused an initial hyperglycaemia, within 0.5 h after injection, and this was followed by a marked hypoglycaemia, which was evident without 2 h and was maintained for a 24 h period of study. The hypoglycaemia was accompanied by a marked reduction in liver glycogen (about 80%) within 2 h after injection (Figure 1b), which was maintained for 4 h, but thereafter there was a progressive increase in glycogen levels such that by 8 h the level was not significantly different from pair-fed controls. Administration of TNF-α also caused a marked hypothermia as measured by rectal body temperature (Figure 1c), which was significantly different from pair-fed controls at 0.5 h, and persisted up to 4 h. This suggests that although there was a considerable drain on blood glucose, it was not being utilised to provide energy for heat generation, or that there was a defect with the thermoregulatory system.

An increased metabolic activity appears not to be responsible for the decrease in blood glucose level since the rate of production of $^{14}CO_2$ from U-$^{14}$C-glucose in TNF-α injected animals was significantly lower than from saline-injected,

**Figure 1** Effect of a single injection of TNF-α (7.5 × $10^7$ U $kg^{-1}$) on (**a**) blood glucose, (**b**) liver glycogen and (**c**) rectal body temperature of female NMRI mice. Each point represents the mean ± s.e.m. of 5–10 animals. $*P < 0.001$ from control, pair-fed animals by Student's $t$ test. Closed symbols TNF-α treated animals, open symbols control.

pair-fed controls (Figure 2a). In contrast production of $^{14}CO_2$ from U-$^{14}$C-palmitic acid in TNF-α injected animals did not differ from controls over a 24 h period (Figure 2b). The decrease in glucose oxidation may be due to the reduced uptake of glucose into muscle (Table I, Figure 4) in TNF-α treated animals. The blood levels of pyruvate, lactate or alanine did not change after TNF-α administration over an 8 h period when compared with saline-injected, pair-fed controls. However, by 24 h lactate levels were significantly higher in TNF-α treated animals (2.6 ± 0.1 mM and 1.9 ± 0.4 mM; $P < 0.05$ by Student's *t* test).

Glucose utilisation by different tissues was investigated *in vivo* by the 2-deoxyglucose tracer method (Meszaros *et al.*, 1987*a*,*b*; Ferre *et al.*, 1985). Blood glucose levels in TNF-α treated animals, that had previously been starved overnight, were significantly lower than controls up to 45 min after treatment (Figure 3a), although there was no difference in the rate of disappearance of the label from 2-deoxy-D-2,6-$^3$H-glucose ($^3$H-2DG) (Figure 3b) or of 2-1-$^{14}$C-deoxy-D-glucose ($^{14}$C-2DG) (Figure 3c) between the two groups. The tissue glucose metabolic rate (*Rg*) of control and TNF-α injected animals is given in Table I. The calculated lumped constant for the liver was 0.45, which was close to the values previously reported for other tissues (Meszares *et al.*, 1987*b*; Ferre *et al.*, 1985) and so this value was utilised to calculate the *Rg* values for all tissues. This shows that 2 h following TNF-α injection the *Rg* values in colon, liver, kidney and spleen were increased by 520, 340, 36 and 25% respectively while the *Rg* values in thigh and gastrocnemius muscles were decreased by 29 and 31% respectively. However, when calculated on a whole organ basis it can be seen that the major

**Table I** Tissue glucose metabolic rate (*Rg*) 2 h after TNF-α ($7.5 \times 10^7$ U $kg^{-1}$) administration[a]

| *Tissue* | *Control* | *TNF-α* |
|---|---|---|
| Liver | 80 ± 4 | 248 ± 15[d] |
| Brain | 2039 ± 105 | 1875 ± 134 |
| Spleen | 1157 ± 40 | 1446 ± 77[d] |
| Kidney | 413 ± 61 | 561 ± 33[c] |
| Pancreas | 661 ± 85 | 544 ± 33 |
| Thigh | 1012 ± 106 | 721 ± 97[b] |
| Gastrocnemius | 1309 ± 142 | 906 ± 121[c] |
| Diaphragm | 155 ± 24 | 206 ± 29 |
| Lung | 1358 ± 203 | 1200 ± 75 |
| Stomach | 249 ± 45 | 176 ± 10 |
| Colon | 26 ± 4 | 134 ± 43[d] |

[a]*Rg* values are expressed as nmol glucose $g^{-1}$ $min^{-1}$ and are given as mean ± s.e.m., $n = 6$; [b]$P < 0.01$ from controls by Student's *t* test; [c]$P < 0.005$ from controls by Student's *t* test; [d]$P < 0.001$ from controls by Student's *t* test.

contributor to an increased glucose utilisation, which may be responsible for the decrease in blood glucose levels after TNF-α administration, is the liver (Figure 4). The magnitude of the contribution of glucose utilisation by the various organs depends on both the increase in *Rg* value and the size of a particular organ. Thus the contribution of the colon to the total body increase in glucose consumption was modest, although this organ showed the largest increase in *Rg* value after TNF-α administration, because of the low contribution to the total body mass.

To investigate whether TNF-α administration altered the retention of 2DGP in the various organs we applied a sequential double labelling technique (Meszaros *et al.*, 1987*a*) followed by an analysis of the two labels in 2DGP. Since

**Figure 2** Effect of TNF-α ($7.5 \times 10^7$ U $kg^{-1}$) on the production of $^{14}CO_2$ from D-U-$^{14}$C-glucose (**a**) or U-$^{14}$C-palmitate (**b**). Both saline-injected pair-fed animals (◘) and TNF-α (◆) injected animals were administered 50 μCi $kg^{-1}$ of the radioisotope immediately after the first injection and the production of $^{14}CO_2$ was determined as described in methods. Each point is the mean ± s.e.m. for 5 animals. **$P < 0.05$ and *$P < 0.001$ from controls by Student's *t* test.

**Figure 3** Plasma glucose concentration (**a**) and the disappearance of $^3$H-2DG (**b**) or $^{14}$C-2DG (**c**) from the blood in control (open symbols) and TNF-α (closed symbols) treated animals. One hour after TNF-α administration ($7.5 \times 10^7$ U $kg^{-1}$) animals were injected with 50 μCi $kg^{-1}$ of $^3$H-2DG and 35 minutes later with 5 μCi $kg^{-1}$ $^{14}$C-2DG and serial blood samples were removed at time intervals. Each point represents the mean ± s.e.m. of 5–10 animals.

**Figure 4** Glucose utilisation rates of different organs of the mouse 2 h after infusion with saline (hatched boxes) or $7.5 \times 10^7$ U kg$^{-1}$ of TNF-α (closed boxes). The results are expressed as mean ± s.e.m. for 6 animals and were based on organ weights and *Rg* values (Table I). A, liver; B, brain; C, spleen; D, kidney; E, pancreas; F, thigh muscle; G, gastrocnemius muscle; H, diaphragm, I, lung; J, stomach; K, colon.

there was a marked initial decay of the precursor in the blood the bulk of the 2-$^3$H-DGP was synthesised in the tissues during the initial 35 min of the labelling period while 2-$^{14}$C-DGP was formed during the second 25 min period and the $^3$H/$^{14}$C ratio of tissue 2DGP was measured at the end of the experiment. Loss of 2DGP from the tissue would affect the $^3$H component of the ratio more than the $^{14}$C component and therefore the $^3$H/$^{14}$C ratio of 2DGP in the tissues was a measure of the retention of 2DGP, i.e. a low ratio indicated a high rate of loss. In these experiments the amount of $^3$H radioactivity administered was 10 times higher than the $^{14}$C and thus the $^3$H/$^{14}$C ratio would be expected to be near 10. This was true for all control tissues except for brain and kidney, which were much lower, and diaphragm, which was much higher (Table II). The lower ratio for brain has previously been reported (Meszaros *et al.*, 1987*a*) and arises from an increased rate of loss of 2DGP from this tissue. The higher ratio for diaphragm indicates that the concentration of 2-$^3$H-DGP was still increasing over the second period. However, after TNF-α administration the $^3$H/$^{14}$C ratios were significantly lower in liver, brain, spleen and lungs, indicating an increased rate of loss of 2DGP from these tissues (Table II).

Since the increased glucose utilisation after TNF-α administration is not associated with an increased respiratory $CO_2$ production (Figure 2a) or an increased blood lactate, alanine or pyruvate level, it suggests increased anabolic reactions. The plasma levels of both FFA (Figure 5a) and triglycerides (Figure 5b) were increased markedly 2–8 h after a single injection of TNF-α, while blood levels of both acetoacetate (Figure 5c) and 3-hydroxybutyrate (Figure 5d) were increased by 2 h and remained elevated for the 24 h period study. This suggests that the increased glucose con-

**Figure 5** Effect of a single injection of TNF-α ($7.5 \times 10^7$ U kg$^{-1}$) on (**a**) plasma FFA, (**b**) plasma triglyceride, (**c**) blood acetoacetate, (**d**) blood 3-hydroxybutyrate concentrations of female NMRI mice. Each point represents the mean ± s.e.m. of 5–6 animals ***$P < 0.05$, **$P < 0.01$, *$P < 0.001$ from control by Student's *t* test. Closed symbols TNF-α treated animals, open symbols control.

sumption by the liver in TNF-α treated animals may be utilised for the biosynthesis of lipids.

To investigate this possibility the conversion of U-$^{14}$C-glucose into lipids in various organs was determined after TNF-α administration. The results in Figure 6 show the effect on liver, spleen, colon, adipose tissue and plasma. The results are expressed as total organ synthesis since this might

**Table II** Labelling of 2DGP in tissues after sequential administration of 2-$^3$H-DG and 2-$^{14}$C-DG[a]

| | *Control d.p.m. g$^{-1}$ tissue* | | | *TNF-α d.p.m. g$^{-1}$ tissue* | | |
|---|---|---|---|---|---|---|
| *Tissue* | *$^3$H-2DGP* | *$^{14}$C-2DGP* | *$^3$H/$^{14}$C* | *$^3$H-2DGP* | *$^{14}$C-2DGP* | *$^3$H/$^{14}$C* |
| Liver | 2620 ± 290 | 180 ± 37 | 13.8 ± 1.8 | 8183 ± 49 | 1100 ± 110 | 7.6 ± 0.6[c] |
| Brain | 67283 ± 3455 | 9900 ± 808 | 7.1 ± 0.3 | 61880 ± 4408 | 10520 ± 863 | 5.9 ± 0.6[c] |
| Spleen | 38167 ± 1319 | 3420 ± 188 | 11.0 ± 0.4 | 47700 ± 2527 | 5933 ± 196 | 8.1 ± 0.4[c] |
| Kidney | 13630 ± 2000 | 2340 ± 350 | 5.9 ± 0.5 | 18520 ± 1100 | 2750 ± 100 | 6.8 ± 0.6 |
| Pancreas | 21800 ± 2800 | 1500 ± 200 | 12.2 ± 1.4 | 17950 ± 1100 | 1870 ± 110 | 9.7 ± 0.5 |
| Thigh | 33400 ± 3500 | 3100 ± 400 | 10.3 ± 1.5 | 23800 ± 3200 | 2720 ± 400 | 9.2 ± 1.3 |
| Gastrocnemius | 43200 ± 4700 | 3880 ± 700 | 9.1 ± 0.8 | 29900 ± 4000 | 3550 ± 330 | 8.4 ± 1.1 |
| Diaphragm | 5110 ± 800 | 340 ± 50 | 18.4 ± 4.1 | 6800 ± 970 | 498 ± 29 | 11.6 ± 2.6 |
| Lungs | 44800 ± 6700 | 4220 ± 840 | 11.6 ± 1.8 | 39600 ± 2490 | 4750 ± 310 | 8.4 ± 0.2[b] |
| Stomach | 8200 ± 1500 | 700 ± 60 | 11.6 ± 1.9 | 5810 ± 330 | 740 ± 120 | 8.9 ± 1.6 |
| Colon | 858 ± 130 | 128 ± 28 | 6.6 ± 2.1 | 4473 ± 1425 | 804 ± 107 | 7.5 ± 2.2 |

[a]Results are mean ± s.e.m., $n = 5–10$; [b]$P < 0.05$ from control by Student's *t* test; [c]$P < 0.001$ from control by Student's *t* test.

**Figure 6** Effect of a single injection of TNF-α ($7.5 \times 10^7$ U $kg^{-1}$) on the conversion of U-$^{14}$C-glucose into lipids in adipose tissue, spleen, blood, liver and colon 2 h (A and B) and 3 h (C and D) in control (A and C) and TNF-α (B and D) treated animals. Animals were administered U-$^{14}$C-glucose (250 μ Ci $kg^{-1}$) and the conversion to $^{14}$C lipids was determined as described in methods. ***$P < 0.05$, **$P < 0.01$, *$P < 0.001$ from control by Student's *t* test. $n = 6$.

be expected to be of greater significance to total body lipid homeostasis. Thus, 2 h after TNF-α administration conversion of glucose into lipids in the liver is more than doubled and this increase is reflected in an increased plasma level of $^{14}$C lipid and an increased synthesis in adipose tissue. At 3 h after TNF-α administration the rate of conversion of $^{14}$C glucose into lipid in the liver was still increased, but the level in plasma was five times that of controls and the level in adipose tissue about four times that of controls. Small increases were also observed in the spleen, but not in the colon at these times. These results suggests that TNF-α administration produces a severe hypoglycaemia in order to serve an increased lipogenesis in liver and adipose tissue.

### Discussion

Acute administration of TNF-α to female NMRI mice has previously been shown to result in a dose-related weight loss, which is directly proportional to a reduction in food and water intake (Mahony *et al.*, 1988; Mahony & Tisdale, 1988). Intracerebroventicular microinfusion of TNF-α has also been shown to suppress food intake in rats (Plata-Salaman, 1988) possibly by inhibition by glucose-sensitive neurons in the lateral hypothalamic area. Profound changes in blood glucose levels occur, which are more severe than we have observed in a tumour-bearing cachexia model (MAC16) (Mahony *et al.*, 1988). An initial hyperglycaemia following TNF-α administration is followed by a dramatic reduction in blood glucose levels below that observed in saline-injected, pair-fed controls, suggesting that the decrease in blood glucose levels is more related to the TNF-α than to the drop in food intake. Similar results have been reported in experimentally induced endotoxic shock (Filkins, 1984). A reduction in lipid absorption from the gastrointestinal tract has been observed following TNF-α administration (Evans & Williamson, 1988) and a reduced nutrient absorption may lead to the sustained hypoglycaemia effect. Concomitant with the reduction in blood glucose levels liver glycogen is reduced by about 80% within 2 h after TNF-α administration and remains below control values up to 8 h. The effect of TNF-α on hepatic glycogen levels may be mediated via increased circulatory levels of glucagon or catecholamines, which have been shown to be increased, together with corticosterone levels, after TNF-α administration (Warren *et al.*, 1987*a*; Bagby *et al.*, 1988) correlating with an increased glucose rate of appearance (Bagby *et al.*, 1988). Previous studies have shown no effect of TNF-α on glycogenolysis, gluconeogenesis or ketogenesis in isolated rat hepatocytes in short term incubations (Rofe *et al.*, 1987) suggesting that the *in vivo* effects may be mediated by secondary modifiers. Although blood lactate levels have been reported to be elevated 3–5-fold after TNF-α administration (Tracey *et al.*, 1986) no increase in blood lactate or pyruvate were observed over short time intervals in this study and no change in blood alanine levels. In humans TNF-α has been shown to cause an increased release of the gluconeogenic amino acids alanine and glutamine from forearm muscle and increase the uptake into other tissues (Warren *et al.*, 1987*b*), while in the rat TNF-α has been shown to have no effect on skeletal protein balance (Kettlehut & Goldberg, 1988). The plasma concentrations of both acetoacetate and 3-hydroxybutyrate were elevated about 2-fold within 2 h after TNF-α administration. Such elevations, which normally occur in prolonged starvation, were not due to the reduction in food intake, since they were not observed in pair-fed animals, and suggest elevated production of acetyl CoA.

Despite the apparent drain on blood glucose levels conversion of glucose into $CO_2$ is reduced after TNF-α administration when compared with pair-fed animals, although production of $^{14}CO_2$ from U $^{14}$C-palmitate is not affected. This again suggests that the reduction of blood glucose is not due to oxidative metabolism and probably arises from an increase in anabolic reactions.

In order to determine the organs responsible for increased glucose consumption after TNF-α administration we applied the 2-deoxyglucose tracer technique. For these experiments animals were starved overnight, which itself reduced plasma glucose to almost the TNF-α treated level (Figure 3a), although it did not prevent TNF-α induced changes in glucose consumption. Previous studies in rats (Meszaros *et al.*, 1987*b*) have shown an increased glucose utilisation by spleen, liver, kidney, skin, diaphragm, lung and ileum after TNF-α administration. When calculated on a whole animal basis the major contributor to the increased glucose utilisation after TNF-α administration in this study was seen to be the liver because of the greater size of this organ. The rate of turnover of glucose using a double isotope labelling technique was also shown to be significantly elevated after TNF-α administration.

Since the elevated glucose consumption is not due to conversion into $CO_2$ or lactate it suggests an enhanced lipogenesis. An enhanced hepatic fatty acid and sterol synthesis has been observed after administration of TNF-α to the rat (Feingold *et al.*, 1987), and the increase in circulating lipid levels after TNF-α administration can occur in the absence of a TNF-induced inhibition of adipose tissue lipoprotein lipase activity (Feingold *et al.*, 1989; Chajek-Shaul *et al.*, 1989). Using U-$^{14}$C-glucose we have shown an elevated conversion to lipids in the liver, adipose tissue and plasma of animals after TNF-α administration. This suggests that the hyperlipidaemia and accumulation of FFA in the plasma after TNF-α administration arise from an increased hepatic output. The parallelism between plasma FFA and triglycerides suggests that the increased triglyceride concentration in plasma is due to esterification of FFA by the liver and secretion as VLDL.

In conclusion our studies demonstrated that administration of TNF-α produces a complex series of metabolic changes, which may represent a co-ordinated metabolic response to infection or cachexia but which seem to be independent of the effect on food and water intake. These conclusions apply to the early effects of TNF-α and the action may be different at later time points.

This work has been supported by a grant from the Cancer Research Campaign. S.M.M. gratefully acknowledges the receipt of a research studentship from the SERC.

### References

BAGBY, G.J., LANG, C.H., HARGROVE, D.M., THOMPSON, J.J., WILSON, L.A. & SPITZER, J.J. (1988). Glucose kinetics in rats infused with endotoxin-induced monokines or tumor necrosis factor. *Circulatory Shock*, **24**, 111.

BEUTLER, B., GREENWALD, D., HULMES, J.D. & 5 others (1985*b*). Identity of tumour necrosis factor and the macrophage-secreted factor cachectin. *Nature*, **316**, 552.

BEUTLER, B., MAHONY, J., LE TRANG, N., PEKALA, P. & CERAMI, A. (1985*a*). Purification of cachectin, a lipoprotein lipase-suppressing hormone secreted by endotoxin-induced RAW264.7 cells. *J. Exp. Med.*, **161**, 981.

CERAMI, A., IKEDA, Y., LE TRANG, N., HOTEZ, P.J. & BEUTLER, B. (1985). Weight loss associated with an endotoxin-induced mediator from peritoneal macrophages: the role of cachectin (tumor necrosis factor). *Immunol. Lett.*, **11**, 173.

CHAJEK-SHAUL, T., FRIEDMAN, G., STEIN, O., SHILONI, E., ETIENNE, J. & STEIN, Y. (1989). Mechanism of the hypertriglyceridemia induced by tumor necrosis factor administration to rats. *Biochim. Biophys. Acta.*, **1001**, 316.

CZOK, R. & LAMPRECHT, W. (1974). Pyruvate, Phosphoenolpyruvate and D-glycerate-2-phosphate. In *Methods of Enzymatic Analysis, 4*, Bergmeyer, H.U. (ed.) p. 1464. Academic Press: New York.

EVANS, R.D. & WILLIAMSON, D.H. (1988). Tumour necrosis factor (cachectin) mimics some of the effect of tumour growth on the disposal of a [$^{14}$C] lipid load in virgin, lactating and litter-removed rats. *Biochem. J.*, **256**, 1055.

FEINGOLD, K.R., GRUNFELD, C., MOSER, A.H., LEAR, S.R. & HUANG, B.-J. (1987). Tumor necrosis factor-alpha stimulates hepatic lipogenesis in the rat *in vivo*. *J. Clin. Invest.*, **80**, 184.

FEINGOLD, K.R., SOUED, M., STAPRANS, I. & 6 others (1989). Effect of tumour necrosis factor (TNF) on lipid metabolism in the diabetic rat. Evidence that inhibition of adipose tissue lipoprotein lipase activity is not required for TNF-induced hyperlipidemia. *J. Clin. Invest.*, **83**, 1116.

FERRE, P., LETURQUE, A., BUMOL, A.-P., PENICAUD, L. & GIRAD, J. (1985). A method to quantify glucose utilization *in vivo* in skeletal muscle and white adipose tissue of the anaesthetized rat. *Biochem. J.*, **228**, 103.

FERRE, P., PEGORIER, J.-P., MARLISS, E.B. & GIRARD, J.R. (1978). Influence of exogenous fat and gluconeogenic substrates on glucose homeostasis in the newborn rat. *Am. J. Physiol.*, **234**, E129.

FILKINS, J.P. (1984). Reticuloendothelial system function and glucose-insulin dyshomeostasis in sepsis. *Am. J. Emerg. Med.*, **2**, 70.

GUTMAN, I. & WAHLEFIELD, A.W. (1974). L-(+)-Lactate determination with lactate dehydrogenase and NAD. In *Methods of Enzymatic Analysis, 4*, Bergmeyer, H.U. (ed.) p. 1463. Academic Press: New York.

KEPPLER, D. & DECKER, K. (1974). Glycogen; determination with amyloglucosidase. In *Methods of Enzymatic Analysis, 3*, Bergmeyer, H.U. (ed.) p. 1127. Academic Press: New York.

KETTLEHUT, I.C. & GOLDBERG, A.L. (1988). Tumor necrosis factor can induce fever in rats without activating protein breakdown in muscle or lipolysis in adipose tissue. *J. Clin. Invest.*, **81**, 1384.

MAHONY, S.M., BECK, S.A. & TISDALE, M.J. (1988). Comparison of weight loss induced by recombinant tumour necrosis factor with that produced by a cachexia-inducing tumour. *Br. J. Cancer*, **57**, 385.

MAHONY, S.M. & TISDALE, M.J. (1988). Induction of weight loss and metabolic alterations by human recombinant tumour necrosis factor. *Br. J. Cancer*, **58**, 345.

MELLANBY, J. & WILLIAMSON, D.H. (1974). Acetoacetate. In *Methods of Enzymatic Analysis, 4*, Bergmeyer, H.U. (ed.) p. 1840. Academic Press: New York.

MESZAROS, K., BAGBY, G., LANG, C. & SPITZER, J.J. (1987*B*). Increased uptake and phosphorylation of 2-deoxyglucose by skeletal muscles in endotoxin-treated rats. *Am. J. Physiol.*, **253**, 33.

MESZAROS, K., LANG, C.H., BAGBY, G.J. & SPITZER, J.J. (1987*b*). Tumor necrosis factor increases *in vivo* glucose utilization of macrophage-rich tissues. *Biochem. Biophys. Res. Commun.*, **149**, 1.

OLIFF, A., DEFO-JONES, D., BOYER, M. & 5 others (1987). Tumors secreting human TNF/cachectin induce cachexia in mice. *Cell*, **50**, 555.

PLATA-SALAMAN, C.R., OOMURA, Y. & KAI, Y. (1988). Tumour necrosis factor and interleukin-1: suppression of food intake by direct action in the central nervous system. *Brain Res.*, **448**, 106.

ROFE, A.M., CONYERS, R.A.J., BAIS, R., GAMBLE, J.R. & VADAS, M.A. (1987). The effects of recombinant tumour necrosis factor (cachectin) on metabolism in isolated rat adipocyte, hepatocyte and muscle preparations. *Biochem. J.*, **247**, 789.

SHERMAN, M.L., SPRIGGS, D.R., ARTHUR, K.A., IMAMURA, K., FREI, E. III & KUFE, D.W. (1988). Recombinant human tumor necrosis factor administered as a five day continuous infusion in cancer patients: Phase 1 toxicity and effects on lipid metabolism. *J. Clin. Oncol.*, **6**, 344.

SOCHER, S.H., MARTINEZ, D., CRAIG, J.B., KUHN, J.G. & OLIFF, A. (1988). Tumor necrosis factor not detectable in patients with clinical cancer cachexia. *J. Natl Cancer Inst.*, **80**, 595.

STANSBIE, D., BROWNSEY, R.W., CRETTAZ, M. & DENTON, R.M. (1976). Acute effects *in vivo* of anti-insulin serum on rates of fatty acid synthesis and activities of acetyl coenzyme-A carboxylase and pyruvate dehydrogenase in liver and epidydimal adipose tissue of fed rats. *Biochem. J.*, **160**, 413.

TRACEY, K.J., BEUTLER, B., LOWRY, S.F. & 9 others (1986). Shock and tissue injury induced by recombinant human cachectin. *Science*, **234**, 470.

TRACEY, K.J., FONG, Y., HESSE, D.G. & 5 others (1987). Anti-cachectin/TNF monoclonal antibodies prevent septic shock during lethal bacteraemia. *Nature*, **330**, 662.

TRACEY, K.J., WEI, H., MANOGUE, K.R. & 8 others (1988). Cachectin/Tumor necrosis factor induces cachexia, anaemia and inflammation. *J. Exp. Med.*, **167**, 1211.

WARREN, R.S., DONNER, D.B., STARNES, H.F. Jr & BRENNAN, M.F. (1987*a*). Modulation of endogenous hormone action by recombinant human tumor necrosis factor. *Proc. Natl Acad. Sci. USA*, **84**, 8619.

WARREN, R.S., STARNES, F. Jr, GABRILOVE, J.L., OETTGEN, H.F. & BRENNAN, M.F. (1987*b*). The acute metabolic effects of tumor necrosis factor administration in humans. *Arg. Surg.*, **122**, 1396.

WILLIAMSON, D.H. (1974). L-Alanine determination with alanine dehydrogenase. In *Methods of Enzymatic Analysis, 4*, Bergmeyer, H.U. (ed.) p. 1679. Academic Press: New York.

WILLIAMSON, D.H. & MELLANBY, J. (1974). D(−)3-Hydroxybutyrate. In *Methods of Enzymatic Analysis, 4*, Bergmeyer, H.U. (ed.) p. 1836. Academic Press: New York.